# SUPPORT Tools for evidence-informed health Policymaking (STP) 17: Dealing with insufficient research evidence

**DOI:** 10.1186/1478-4505-7-S1-S17

**Published:** 2009-12-16

**Authors:** Andrew D Oxman, John N Lavis, Atle Fretheim, Simon Lewin

**Affiliations:** 1 Norwegian Knowledge Centre for the Health Services, P.O. Box 7004, St. Olavs plass, N-0130 Oslo, Norway; 2 Centre for Health Economics and Policy Analysis, Department of Clinical Epidemiology and Biostatistics, and Department of Political Science, McMaster University, 1200 Main St. West, HSC-2D3, Hamilton, ON, Canada, L8N 3Z5; 3 Norwegian Knowledge Centre for the Health Services, P.O. Box 7004, St. Olavs plass, N-0130 Oslo, Norway; Section for International Health, Institute of General Practice and Community Medicine, Faculty of Medicine, University of Oslo, Norway; 4 Norwegian Knowledge Centre for the Health Services, P.O. Box 7004, St. Olavs plass, N-0130 Oslo, Norway; Health Systems Research Unit, Medical Research Council of South Africa

## Abstract

*This article is part of a series written for people responsible for making decisions about health policies and programmes and for those who support these decision makers*.

In this article, we address the issue of decision making in situations in which there is insufficient evidence at hand. Policymakers often have insufficient evidence to know with certainty what the impacts of a health policy or programme option will be, but they must still make decisions. We suggest four questions that can be considered when there may be insufficient evidence to be confident about the impacts of implementing an option. These are: 1. Is there a systematic review of the impacts of the option? 2. Has inconclusive evidence been misinterpreted as evidence of no effect? 3. Is it possible to be confident about a decision despite a lack of evidence? 4. Is the option potentially harmful, ineffective or not worth the cost?

## About STP

*This article is part of a series written for people responsible for making decisions about health policies and programmes and for those who support these decision makers. The series is intended to help such people ensure that their decisions are well informed by the best available research evidence. The SUPPORT tools and the ways in which they can be used are described in more detail in the Introduction to this series *[1]. *A glossary for the entire series is attached to each article (see Additional File *1*). Links to Spanish, Portuguese, French and Chinese translations of this series can be found on the SUPPORT website *http://www.support-collaboration.org. *Feedback about how to improve the tools in this series is welcome and should be sent to*: STP@nokc.no.

## Scenario

*The Ministry of Health is considering strategies to recruit and retain health professionals in underserved rural areas. You have been asked to advise the Minister of Health about these strategies. You have found many articles describing strategies that have been used in other settings, but no reliable evaluations of the impacts of such strategies *[2].

## Background

In this article, we present five questions that policymakers and those who support them can ask when considering scenarios in which there may be insufficient evidence to inform judgements about the impacts of policy and programme options.

It is unrealistic to assume that one can predict the impacts of a health policy or programme with certainty. Many governance, financial and delivery arrangements have not been rigorously evaluated. Neither have many of the programmes, services and drugs that these arrangements support. But policymakers must still make decisions regardless of the availability (or paucity) of evidence to inform such decisions.

In this article, we focus on decision making undertaken in instances in which there is insufficient evidence available to be able to know whether an option will have the impacts intended, or whether it may have unintended (and undesirable) impacts. Common mistakes made when there is insufficient evidence at hand include making assumptions about the evidence without a systematic review, confusing a lack of evidence with evidence of no effect, assuming that insufficient evidence necessarily implies uncertainty about a decision, and the assumption that it is politically expedient to feign certainty. We present four questions in this article that can help to avoid these.

## Questions to consider

If there is insufficient evidence at hand to allow one to be confident about the impacts of implementing a policy or programme option, the following questions can be considered:

1. Is there a systematic review of the impacts of the option?

2. Has inconclusive evidence been misinterpreted as evidence of no effect?

3. Is it possible to be confident about a decision despite a lack of evidence?

4. Is the option potentially harmful, ineffective or not worth the cost?

### 1. Is there a systematic review of the impacts of the option?

The first step in addressing a perceived lack of evidence is to find out what evidence *is *available. It is risky to make assumptions about the availability of evidence without referring to systematic reviews. Considerations related to finding and critically appraising systematic reviews are addressed in Articles 5 and 6 in this series [3,4].

For many questions related to health systems it is not possible to find relevant and up-to-date systematic reviews. There is widespread recognition, for example, that health workers are critical to achieving the Millennium Development Goals (MDGs) and other health goals. Yet despite this, an overview of systematic reviews of options to address human resources for health found only a small amount of high-quality, synthesised research evidence regarding the effects of a few options for the improvement of human resources for health [5]. Other overviews of reviews have found similar gaps [e.g. [6]]. A lack of systematic reviews may not necessarily reflect a lack of evidence. But under such circumstances it is difficult for policymakers to know what evidence is available (see Table 1, for example).

**Table 1:** An independent inquiry into inequalities in health - an example of the need for up-to-date systematic reviews to know what evidence there is

In 1997, the incoming British Labour government was keen to reduce inequalities in health. To do this, it set about obtaining advice from the public health community about how to reduce inequalities, but clear limits were set about what advice it would find acceptable. The government wanted the advice quickly but stipulated that the advice had to be backed by evidence, in keeping with the government's expressed desire that public policy should be based on evidence [31]. The public health and other communities responded enthusiastically. A considerable amount of material was produced by, and for, the inquiry and many recommendations were made [32].
Subsequent reviews of the recommendations, however, found little evidence for the likely or actual effectiveness of many of the recommendations [32]. There was also a striking lack of adequate searches for relevant evidence or attempts to avoid bias in the way information was identified, appraised, and used.
This is not to suggest that governments cannot develop or implement policies that lack the support of unequivocal evidence. A lack of evidence does make it difficult, however, for them to decide on priorities. The readiness of researchers to recommend policies while knowing little about the likely effectiveness makes this more difficult still.
The task of this particular inquiry in the United Kingdom would have been easier if up-to-date systematic reviews had been available. Further, a system to ensure that the inquiry's recommendations would be reviewed regularly as new information and evidence emerged from updated systematic reviews, would have helped to ensure that adjustments in policies could have been made. This could also have helped to avoid similar future difficulties when similar inquiries were undertaken or similar policies considered in other jurisdictions. International networks such as The Cochrane Collaboration http://www.cochrane.org (which focuses on healthcare) and the Campbell Collaboration http://www.campbellcollaboration.org (which focuses on education, crime and justice, and social welfare) have structures for preparing and keeping systematic reviews up-to-date, and these can facilitate the more effective use of evidence.
The investment of public resources in primary research has been substantial and remains so. But the returns remain far less than might otherwise have been expected, and the results scattered rather than synthesised. People faced with tasks and timescales similar to those of the British inquiry would be assisted greatly if up-to-date systematic reviews were more readily available. In terms of developing health policies and programmes, there are no unequivocal answers to the question "What works?" A systematic review is the best starting point for finding out what is known.

Rapid assessments may need to be undertaken when time or resources are limited. These assessments should be transparent about the methods used, as well as any important methodological limitations or related uncertainties. They should also address the need for, and urgency of, undertaking a full systematic review at a later date [7]. Consideration should also be given to commissioning a new review whenever a relevant, up-to-date review of good quality is unavailable. Appropriate processes should be used, including setting priorities for systematic reviews [8]. Building and strengthening international collaborations, such as the Cochrane Collaboration http://www.cochrane.org, can help to avoid unnecessary duplications of effort involved in producing systematic reviews and help to ensure that up-to-date reviews are more readily available.

### 2. Has inconclusive evidence been misinterpreted as evidence of no effect?

Another common mistake made in instances when evidence is inconclusive is the confusion of a lack of evidence of an effect with 'evidence of no effect' [9]. It is wrong to claim that inconclusive evidence shows that a policy or programme has had 'no effect'. 'Statistical significance' should *not *be confused with importance.

**Figure 1:**
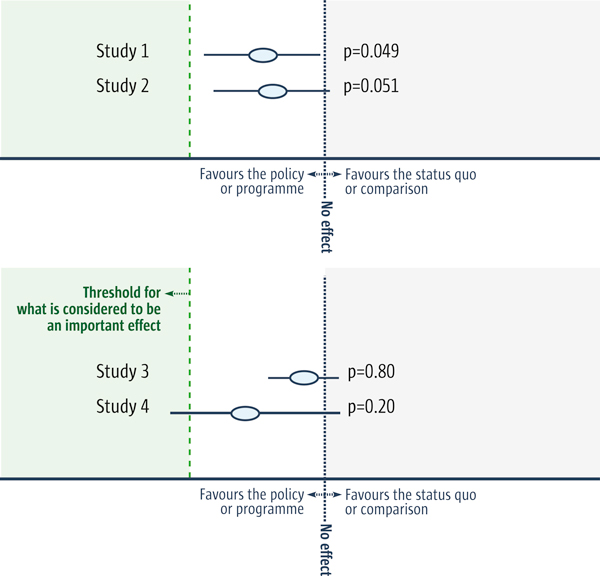
**Two problems with classifying results as 'statistically non-significant' or 'negative'**. The blue dots in the Figure above indicate the estimated effect for each study and the horizontal lines indicate the 95% confidence intervals. A 95% confidence interval means that we can be 95% confident that the true size of the effect is between the lower and upper confidence limit (the two ends of the horizontal lines). Conversely, there is a 5% chance that the true effect is outside this range.

When results are not 'statistically significant' it cannot be assumed that there was no impact. Typically a cut-off of 5% is used to indicate statistical significance. This means that the results are considered to be 'statistically non-significant' if the analysis shows that differences as large as (or larger than) the observed difference would be expected to occur by chance *more *than one out of twenty times (p ≥0.05). There are, however, two problems with this assumption. Firstly, the cut-off point of 5% is arbitrary. Secondly, 'statistically non-significant' results (often mislabelled as 'negative'), might or might not be inconclusive. Table 2 contains a further discussion of this point and Figure 1 illustrates how the use of the term 'statistically non-significant' or 'negative' can be misleading.

**Table 2:** 'Statistical non-significance'

Figure 1 illustrates two problems that arise when results are classified as 'statistically non-significant' or 'negative':
1. *The classification is based on an arbitrary cut-off*. The results of Study 1, for example, are marginally different from the results of Study 2. But by using the conventional cut-off of P < 0.05, the results of Study 1 are ranked as 'statistically significant' and the results of Study 2 as 'statistically non-significant'.
2. *'Statistically non-significant' results may or may not be inconclusive*. If the short green vertical line in the figure below indicates the smallest effect considered important, the results for Study 3 would be conclusive, since an important impact is highly unlikely. The results for Study 4 would be categorised as 'inconclusive' since it is not unlikely that there would be an important impact (the 95% confidence interval crosses the threshold for what is considered to be an important effect). Both results, however, might be regarded as 'statistically non-significant' or 'negative'.

Trends that are 'positive' (i.e. in favour of an option) but 'statistically non-significant' are often described as 'promising' and this can also be misleading. 'Negative' trends of the same magnitude, in contrast, are not typically described as 'warning signs'.

Policymakers should be aware that researchers commonly make these mistakes. To avoid being misled, they should be watchful for misinterpretations of statistical significance.

### 3. Is it possible to be confident about a decision despite a lack of evidence?

Some policymakers may agree with Charlie Brown, who claimed: "I am always certain if it is a matter of opinion" But most would agree that high-quality evidence provides a better basis for being confident about decisions. Nevertheless, there may be good reasons for being confident about a decision even when there is a lack of evidence. There is very low-quality evidence, for example, that giving aspirin to children with influenza or chicken pox may cause Reye's syndrome (a rare but deadly condition) [10]. Despite the limitations of this evidence, the US Surgeon General and others have confidently advised against the use of aspirin in these circumstances. This is because of the availability of paracetamol (acetaminophen) as an equally effective and inexpensive alternative which allows children not to be put at risk, even if there is uncertainty about the actual level of the risk itself. Conversely, it may be reasonable to be confident that policies or programmes with high costs and potentially serious adverse effects should *not *be rolled out without a rigorous impact evaluation.

### 4. Is the option potentially harmful, ineffective or not worth the cost?

*"Professional good intentions and plausible theories are insufficient for selecting policies and practices for protecting, promoting and restoring health. Humility and uncertainty are preconditions for unbiased assessments of the effects of the prescriptions and proscriptions of policy makers and practitioners for other people. We will serve the public more responsibly and ethically when research designed to reduce the likelihood that we will be misled by bias and the play of chance has become an expected element of professional and policy making practice, not an optional add-on." *(Iain Chalmers, Editor, the James Lind Library, presentation at the Norwegian Directorate for Health and Social Welfare, 1 September 2003. For a more detailed discussion of these comments see Reference [11])

It is risky not to acknowledge uncertainty for the sake of political expediency. As we noted in Article 1 in this series [12], acknowledging that there is imperfect information to inform policies can reduce political risk because it allows policymakers to set in motion ways to alter course if policies do not work as expected.

As the quote above suggests, good intentions and plausible theories are insufficient when selecting policies and practices. This is true for health systems as well as clinical interventions. Examples of clinical interventions found to be relatively ineffective or harmful after initially being believed to be beneficial and widely used, include:

• High instead of low osmolar rehydration solutions for children with diarrhoea [13]

• Diazepam or phenytoin instead of magnesium sulphate for women with eclampsia [14,15]

• Six or more antenatal care visits instead of four [16]

• Corticosteroids for patients with severe head trauma [17]

• Albumin instead of salt water for resuscitation in critically ill patients [18]

• Hormone replacement therapy to reduce the risk of coronary heart disease and stroke in women [19]

• Electronic mosquito repellents for preventing mosquito bites and malaria infection [20]

All the above interventions were based on underlying theories, indirect evidence, surrogate outcomes, and observational studies: randomised trials subsequently disproved all the underlying assumptions. This supports the assertion (quoted above) that by making rigorous evaluations an *expectation *rather than an *option *for informing decisions about the provision of clinical interventions, the public can be more responsibly and ethically served.

These same concerns apply to health systems and public health interventions. Examples of health systems and public health interventions that have been widely used and advocated, but which may be ineffective and do more harm than good, include the following:

• Educational and community interventions to reduce the risk of teenage pregnancy [21]

• Directly observed therapy for tuberculosis [22]

• User fees for essential medicines [23]

• For-profit instead of not-for-profit private hospitals [24]

• Reducing maldistribution by requiring doctors to spend a minimum number of years in an underserved area before allowing them to specialise [2]

• Some forms of results-based financing or pay-for-performance [25]

• Contracting with the private sector to provide health services [26]

Substantial caution is required before investing scarce resources in policies or programme options requiring large investments that cannot be recouped [27]. If there is important uncertainty about the impacts of such options, a rigorous evaluation (such as a pilot study, for example), can prevent the potential for resource wastage. And while such undertakings may appear to present unnecessary delays, Julio Frenk, the former Minister of Health from Mexico, has noted: "Both politically, in terms of being accountable to those who fund the system, and also ethically, in terms of making sure that you make the best use possible of available resources, evaluation is absolutely critical" [28]. Decisions both in support of an option and those against, may be equally likely to have undesirable consequences if there is insufficient evidence (see Table 3 for an example and further explanation). Informing policymaking by testing a proposed option within a well-designed impact evaluation offers a better approach.

**Table 3:** The consequences of saying "no" or "yes" instead of "only in the context of an evaluation"

All countries face resource constraints. For this reason, in the United Kingdom for example, the National Institute for Health and Clinical Excellence (NICE) officially recognises the principle of recommending that when important uncertainties exist about an intervention's effects, such interventions should only be used in the context of research [27]. Sixteen (approximately 4%) of NICE's technology appraisal recommendations published between 1999 and early 2007 advised the use of a technology only in the context of research. The consequences of getting decisions wrong by either saying "no" or "yes" to a technology without doing so, are summarised below (see Reference [27] for further details).
**The consequence of saying "no" instead of "only in research"**
• Patients are denied access to promising and potentially effective technologies
• There are delays in building the evidence base in key areas, with a resulting negative overall impact on health outcomes
**The consequences of saying "yes" instead of "only in research"**
• Access to unproven and potentially harmful or ineffective interventions is promoted
• Any ongoing or future research in the field is severely hindered. Important questions on effectiveness and cost-effectiveness may never be answered
• Limited resources are wasted
• Having to reverse a "yes" decision in the light of any future evidence compromises credibility and is difficult to implement

When judgements about the effects of options are based on theories, surrogate outcomes, limited observational studies, inadequate impact evaluations, anecdotal experience or analogies, policymakers should be cautious about implementing them (see example in Table 4) [29].

**Table 4:** An example of a potentially ineffective or harmful intervention that has been widely promoted based on insufficient evidence

Effective drugs for tuberculosis have been available since the 1940s. Despite this, two million people continue to die from the disease each year, mostly in low-income countries. People with tuberculosis require treatment that lasts between six to eight months. Many find it difficult to complete their course of treatment and this serves as a major constraint to eradicating the disease. Poor adherence to treatment can lead to prolonged infectiousness, drug resistance, relapses, or even death. Incomplete treatment thus poses a serious risk both to the individual and to communities as a whole.
Directly observed therapy (DOT) seeks to improve the adherence of people to tuberculosis treatment by using health workers, family members, or community members to directly observe patients taking their anti-tuberculosis drugs. DOT is potentially advantageous because adherence may improve when people are closely monitored and there is a social process involving peer pressure. Potential disadvantages include the fact that this treatment moves away from adherence models of communication, with their emphasis on cooperation between patient and provider, back to a traditional medical approach where the patient is a passive recipient of advice and treatment. Also, resource implications for such a policy are substantial, particularly in low- and middle-income countries where the case load may be high. DOT may also make adherence worse if it is rigidly applied in an authoritarian setting, or where people are expected to travel considerable distances to have their treatment supervised.
The World Health Organization (WHO) and others have actively promoted DOT since the 1980s, generally as part of a comprehensive tuberculosis management programme known as DOTS (directly observed therapy, short course), a five-element strategy for the control of tuberculosis. Although the strategy as a whole appears sound, there is substantial uncertainty about DOT as a key element of DOTS. When DOTS was originally launched, the evidence for the effectiveness of DOT came entirely from observational studies and no randomised impact evaluations of DOT had been undertaken. Subsequently, 11 randomised trials have compared DOT with self-administration and found that DOT did not improve adherence, despite the substantial resources required and its other disadvantages [22].

And even if there is little uncertainty about the benefits of an option, there may still be important uncertainty about *other *potentially important consequences, including unintended effects (harms) and costs (see example in Table 5). Policies or programmes with compelling rationales can, in fact, cause harm.

**Table 5:** An example of important uncertainties about potentially important harms

Although there is little doubt that financial incentives, if they are large enough, can change behaviours, they can also cause unintended behaviours. The costs of both the incentives and their administration can also be substantial [25]. Unintended effects of paying for performance (the provision of payment for the attainment of well-defined results) that have been observed include:
• *Unintended behaviours*: Conditional cash transfers (CCT) have caused some mothers to keep their children malnourished in order to retain eligibility. An increase in fertility of between 2% and 4%, noted in another study, may have been due to the fact that only pregnant women were eligible for a CCT subsidy
• *Distortions*: Financial incentives may cause recipients to ignore other important tasks
• *Gaming*: Financial incentives can result in gaming (changes in reporting rather than desired changes in practice)
• *Corruption*: Financial incentives may be stolen or misused, if not adequately managed
• *Cherry-picking*: Performance incentives for providers can influence whether healthcare is accessible to patients by altering how willing healthcare workers or organisations are to care for sicker patients, more disadvantaged populations, or more difficult patients
• *Widening the resource gap between rich and poor*: Performance incentives for providers may widen the resource gap that exists between organisations that serve disadvantaged patients and those that do not
• *Dependency on financial incentives*: Relying on incentives may foster dependency on them. If provider behaviours are not ingrained, they may decline or disappear when the incentives end or new incentives are introduced
• *Demoralisation*: Financial incentives may cause feelings of injustice and demoralisation in instances where, for example, professionals on short-term contracts receive more financial incentives than those who have established long-term practices, or where favouritism is perceived
• *Bureaucratisation*: Results-based financing schemes may have substantial administrative costs associated with monitoring performance and managing disbursement of the financial incentives

For an option that is promising, but for which there is insufficient evidence to be confident about whether it is potentially harmful, ineffective, or not worth the cost, consideration should be given to requiring a well-designed impact evaluation. This can be undertaken either prior to rolling out the policy or programme, or integrated as part of the rollout. We address further considerations regarding monitoring and evaluation in Article 15 of this series [29].

## Conclusion

Most health policies and programmes are complex and they are likely to have multiple effects. Some evidence will almost always be available based on experience with similar policies or programmes in other settings. However, as addressed in Articles 8 and 9 in this series, it is important for policymakers to consider how much confidence to place in such evidence and to assess the applicability of the findings to their own setting [4,30]. Typically, there will be uncertainty about the impacts of policies and programmes on important outcomes. When there is important uncertainty, common mistakes such as those described in this article should be avoided.

## Resources

### Useful documents and further reading

- Chalkidou K, Hoy A, Littlejohns P. Making a decision to wait for more evidence: When the National Institute for Health and Clinical Excellence recommends a technology only in the context of research. J R Soc Med 2007; 100:453-60. http://jrsm.rsmjournals.com/cgi/content/full/100/10/453

- Oxman AD, Bjørndal A, Becerra F, Gonzalez Block MA, Haines A, Hooker Odom C, et al. Helping to ensure well-informed public policy decisions: a framework for mandatory impact evaluation. Lancet. In press.

## Competing interests

The authors declare that they have no competing interests.

## Authors' contributions

ADO prepared the first draft of this article. JNL, AF and SL contributed to drafting and revising it.

## Supplementary Material

Additional file 1 Glosssary Click here for file
